# Factors that could explain the increasing prevalence of type 2 diabetes among adults in a Canadian province: a critical review and analysis

**DOI:** 10.1186/s13098-016-0186-9

**Published:** 2016-11-09

**Authors:** Véronique Thibault, Mathieu Bélanger, Emilie LeBlanc, Lise Babin, Stuart Halpine, Beverly Greene, Michelina Mancuso

**Affiliations:** 1 Faculty of Medicine and Health Sciences, Université de Sherbrooke, 2500 boul. de l’Université, Sherbrooke, QC J1K 2R1 Canada; 2 Centre de formation médicale du Nouveau-Brunswick, 100 Des Aboiteaux St, Moncton, NB E1A 7R1 Canada; 3 Vitalité Health Network, 275 Main Street Suite 600, Bathurst, NB E2A 1A9 Canada; 4 New Brunswick Department of Health, 520 King Street, Fredericton, NB E3B 6G3 Canada; 5 New Brunswick Health Council, 100 des Aboiteaux Street, Suite 2200, Moncton, NB E1A 7R1 Canada; 6 100 rue des Aboiteaux, Pavillon J.-Raymond Frenette, Moncton, NB E1A 3E9 Canada

**Keywords:** Diabetes mellitus, Type 2 diabetes, Epidemiology, Prevalence, Trends, Factors

## Abstract

**Background:**

The prevalence of diabetes has increased since the last decade in New Brunswick. Identifying factors contributing to the increase in diabetes prevalence will help inform an action plan to manage the condition. The objective was to describe factors that could explain the increasing prevalence of type 2 diabetes in New Brunswick since 2001.

**Methods:**

A critical literature review was conducted to identify factors potentially responsible for an increase in prevalence of diabetes. Data from various sources were obtained to draw a repeated cross-sectional (2001–2014) description of these factors concurrently with changes in the prevalence of type 2 diabetes in New Brunswick. Linear regressions, Poisson regressions and Cochran Armitage analysis were used to describe relationships between these factors and time.

**Results:**

Factors identified in the review were summarized in five categories: individual-level risk factors, environmental risk factors, evolution of the disease, detection effect and global changes. The prevalence of type 2 diabetes has increased by 120% between 2001 and 2014. The prevalence of obesity, hypertension, prediabetes, alcohol consumption, immigration and urbanization increased during the study period and the consumption of fruits and vegetables decreased which could represent potential factors of the increasing prevalence of type 2 diabetes. Physical activity, smoking, socioeconomic status and education did not present trends that could explain the increasing prevalence of type 2 diabetes. During the study period, the mortality rate and the conversion rate from prediabetes to diabetes decreased and the incidence rate increased. Suggestion of a detection effect was also present as the number of people tested increased while the HbA_1c_ and the age at detection decreased. Period and birth cohort effect were also noted through a rise in the prevalence of type 2 diabetes across all age groups, but greater increases were observed among the younger cohorts.

**Conclusions:**

This study presents a comprehensive overview of factors potentially responsible for population level changes in prevalence of type 2 diabetes. Recent increases in type 2 diabetes in New Brunswick may be attributable to a combination of some individual-level and environmental risk factors, the detection effect, the evolution of the disease and global changes.

## Background

With 382 million people living with diabetes in 2013 [1], the World Health Organization declared the condition as being epidemic [2]. It is estimated that diabetes will affect 3.7 million Canadians in 2018, making it the principal health challenge of the country [3]. Diabetes is associated with many health complications. Comparing the population with and without diabetes, those with diabetes have a 300% increased risk of being hospitalized [3]. Annual per capita healthcare costs for people with diabetes are three to four times greater than for individuals without diabetes [3]. New Brunswick is one of the provinces in Canada where the prevalence of diabetes is the highest [3]. The prevalence of diabetes is estimated to have increased by 86% in this province between 2000 and 2010 [4].

A population-level increase in prevalence of diabetes may be attributable to a wide range of potential factors [5–7]. Obesity is often seen as the main contributor to an increasing prevalence of diabetes [8–10] but other factors such as ageing, ethnicity, lifestyle (i.e., physical inactivity and energy dense diet), socioeconomic status, education, and urbanization have also been identified as potentially important factors [11–14]. Further findings also suggest that increasing incidence rates of diabetes [15–18] and global changes are other potential explanatory factors (e.g. environmental pollution, obesogenic environment and rapid socioeconomic development) that could affect the entire population [19, 20]. In addition to changes in the prevalence of risk factors, other elements, including increases in screening [10], changes in diagnostic criteria [21], and decreasing mortality rates among individuals with diabetes [15–18, 22–32] could contribute to the rise in prevalence of this condition.

Perhaps due to the wide variety of factors potentially responsible for an increase in prevalence of diabetes, no studies have attempted to present a comprehensive list of factors which could be responsible for population-level changes in prevalence of diabetes. A comprehensive list of factors that contribute to the growing prevalence of diabetes would provide a foundation for population health planning in developing successful strategies to address this epidemic of diabetes. As such, the objectives of this study were (1) to develop a comprehensive list of factors to consider when trying to identify causes of change in prevalence of diabetes in a population and (2) to use this list to describe factors that may be responsible for the recent increase in prevalence of type 2 diabetes in New Brunswick.

## Methods

### Step 1: critical review procedure

A critical review of the literature was conducted to identify all factors potentially involved in explaining changes in the prevalence of diabetes. A critical review includes an extensive research of the literature and an evaluation of its quality without a systematic research approach [33]. This type of review includes a description of identified articles, synthesizes and analyzes the information to develop an hypothesis or a model for further study [33]. The first step of the critical review was to identify studies that aimed to document a wide range of factors explaining changes in the prevalence of diabetes. The following term was used in the PubMed database to identify only review articles: “Review[ptyp]”. Additional terms were also entered at this step to identify any review articles on the topic of diabetes prevalence, trends, or epidemiology: “prevalence” [title/abstract] AND “diabetes/prevention and control “[majr] OR (“Diabetes” [title/abstract] AND “trends” [title/abstract] OR “diabetes/epidemiology “[majr]). The second step of the critical review searched studies that described other factors without restricting to only review articles. At this point, we used this research procedure “prevalence” [title/abstract] AND (“diabetes/prevention and control “[majr]) OR (“Diabetes” [title/abstract] AND “trends” [title/abstract]). From the 3215 articles of the first research procedure and 1907 of the second, 446 were duplicates. Of the 4676 unique articles, we retained six review articles that presented multiple potential determinants of diabetes prevalence. All other articles were scanned to retain any articles (n = 71) which presented other factors not considered in the review articles identified and to provide additional empirical support to the factors identified in the reviews. In total, 77 articles were included in this critical review.

### Step 2: description of potential determinants of diabetes prevalence

#### Data sources and study population

The changes in factors potentially involved in explaining an increase in a population’s prevalence of diabetes identified in the critical review were studied using a descriptive repeated cross sectional analysis approach. De-identified registries of all individuals with diabetes and all individuals with prediabetes were created from New Brunswick Department of Health administrative datasets and were stored in a secured office within the New Brunswick Health Council. Those administrative datasets included the list of all New Brunswickers with provincial healthcare coverage (all Canadian residing in New Brunswick) and a repository of all HbA_1c_ tests conducted between 2001 and 2014. Individuals were identified as having diabetes the first time they had a HbA_1c_ test ≥6.5% (48 mmol/mol) and were excluded from prevalence estimates after they died based on their date of death. Similarly, individuals were identified as having prediabetes the first time they had a HbA_1c_ test between 6.0 (42 mmol/mol) and 6.4% (47 mmol/mol) and were removed from the prediabetes registry if they developed diabetes, or if they had a normal HbA_1c_ test (<6.0%) (<42 mmol/mol), or if they died. Because only type 2 diabetes population was of interest for this study and because it was not possible to differentiate the type of diabetes in the diabetes registry, only those ≥30 years of age at detection were included in the analysis, with no maximum age limit. This approach is used by the New Brunswick Department of Health for their provincial reports and has been used by other authors [6] since diabetes cases diagnosed before this age are more often type 1 diabetes [34]. Other data were obtained from the Canadian Census of Population, the Canadian Community Health Survey, the National Household Survey, the Labour Force Survey and the Income Statistics Division from Statistics Canada. The New Brunswick databases used for this study were completely anonymized and the study was approved by the Institutional Review Board of the Centre hospitalier universitaire de l’Université de Sherbrooke.

### Variables

Prevalence of diabetes and prediabetes were measured annually as the proportion of individuals with diabetes and the proportion of individuals with prediabetes divided by their respective populations at risk during the fiscal year, defined as April 1st to March 31st. The population at risk was determined based on the number of New Brunswickers aged 30 or older recorded from the annual estimates of the population from Statistics Canada. The denominator for incidence rates corresponded to the at-risk population described above minus the prevalent cases and incident cases during the year. Mortality rate corresponded to the number of people with diabetes who died during the fiscal year. The denominator included all people living with diabetes, including the time alive among those who died within the given year. The conversion rate from prediabetes to diabetes was calculated as the number of individuals with prediabetes who developed diabetes during the fiscal year over the number of individuals with prediabetes. Number of people with HbA_1c_ testing was defined annually as the number of people with at least one HbA_1c_ test during the fiscal year. Age at detection was derived from the date at which the first test attained the diagnostic threshold and the documented birth date of the individuals. HbA_1c_ at detection was obtained by averaging the HbA_1c_ value at detection of all incident cases for each fiscal year. The breakdown of the population by age groups was obtained from 2001 to 2014 from annual estimates of the entire New Brunswick population from Statistics Canada. The prevalence of select risk factors was determined from self-reported information available for the New Brunswick population from the Canadian Community Health Survey in 2003, 2005 and annually from 2007 to 2014 for people aged 35 years old and greater. These risk factors included *obesity* (body mass index ≥30, calculated from self-reported height and weight data), *hypertension* (self-reported diagnosis received from a healthcare professional), *consumption of fruits and vegetables* (eating fruits and vegetables usually more than five times per day), *physical activity* (categorized as active or inactive based on the average daily physical activity reported for the 3 previous months), *alcohol consumption* (having drunk more than five glasses of alcohol at one occasion, at least once a month during the last year—note that because of changes in definitions in 2013, only data up to 2012 were considered herein) and *smoking* (smoking cigarettes every day). *Level of education* (number of people who completed high school; number with a university degree) was obtained from the Labour Force Survey from 2001 to 2014. *Socioeconomic status* (percentage of people having low income before taxes) was available from 2001 to 2011 from the Income Statistics Division of Statistics Canada and *urbanization* (people living in a rural area, defined as any territory situated outside population centers which contain a population of at least 1000 person and a density of 400 or more person by square kilometer) was obtained from Census data for 2001, 2006 and 2011. *Ethnicity* (percentage of immigration used as a proxy for ethnicity herein) was also obtained from Census data for 2001 and 2006 and from the National Household Survey for 2011.

### Statistical analysis

Cochran–armitage analyses of trend were used to assess the association between time (fiscal year) and variables represented as yearly prevalence. Poisson regressions were used to assess associations between time and mortality rate and between time and conversion rate from prediabetes to diabetes. Linear regressions were used to assess time trends in number of people who undergone HbA_1c_ testing, the average value of HbA_1c_ and the average age at detection. Assessing the presence of a period effect was done by calculating the prevalence of diabetes across 10 year age groups (30–39, …, 90+) for each fiscal year. Assessing for the presence of a birth cohort effect was also done by calculating the prevalence of diabetes among decade-of-birth-based cohorts (1910–1920, …, 1961–1970) for each fiscal year.

## Results

From 101,519 individuals identified with diabetes in the registry between 2001 and 2014, 97,865 individuals were included because they were diagnosed at 30 years old or older. The prevalence of type 2 diabetes increased by an average of 6.4% per year over this time period, which represents an overall increase of 120% (Table 1). The critical literature review identified several factors that could potentially explain such a change in the prevalence of diabetes at a population level. The factors have been grouped in five categories (Table 2) which are defined below. A description of these factors from 2001 to 2014 in New Brunswick is also presented to better understand how they could have contributed to the rise in prevalence of diabetes in this region. In Table 2, factors for which we found information and which we included in the analyses are presented in italics.

**Table 1:** Description of factors related to the prevalence of type 2 diabetes

	Fiscal year													
	2001	2002	2003	2004	2005	2006	2007	2008	2009	2010	2011	2012	2013	2014
*Prevalence of type 2 diabetes*														
Prevalence of T2D %	6.71	7.03	7.81	8.73	9.78	10.7	11.2	11.6	12.1	12.8	13.5	14.0	14.4	14.9^a^
*Individual*-*level risk factors*														
Median population age (years)	38.2	38.8	39.4	39.9	40.5	41.1	41.6	42.0	42.4	42.7	43.0	43.4	43.9	44.3^a^
Health factors														
Obesity %			22.4		25.3		23.2	25.2	30.6	30.0	27.3	32.5	28.3	28.3^a^
Hypertension %			30.0		34.7		34.8	36.1	37.4	39.3	37.9	39.7	37.0	38.1^a^
Prediabetes %	0.87	1.12	1.63	2.27	3.14	3.87	4.24	4.18	4.67	5.44	6.02	6.14	6.01	6.41^a^
Life style habits														
Fruits and vegetables consumption %			33.1		36.1		38.3	38.7	39.2	36.0	34.7	31.4	35.8	32.5^a^
Moderate and high PA levels %			41.4		41.4		39.7	45.4	44.3	48.6	46.8	48.3	44.0	45.9^a^
Inactive PA levels %			60.3		59.5		61.0	56.9	57.3	53.1	54.6	52.6	56.8	55.3^a^
Smoking habits %			23.8		19.5		24.8	21.4	20.0	19.9	20.3	22.1	21.2	19.8^a^
Alcohol consumption %			18.0		18.0		17.2	18.3	16.8	18.9	22.0	21.0^a^		
Education														
High school %	69.5	70.7	72.1	72.8	73.5	75.0	74.9	76.4	77.2	77.6	76.7	78.3	78.2	80.7^a^
University %	12.9	13.2	13.5	13.8	14.7	15.1	15.7	16.1	16.5	16.6	16.5	16.0	17.7	18.0^a^
Socioeconomic status														
Low income %	13.9	14.8	15	14.4	14.2	14.2	13.9	11.6	10	9.3	9.2^a^			
Ethnicity														
Immigration %	3.1					3.7					3.9^a^			
*Environmental risk factors*														
Urbanization														
Rural area %	49.8					49.1					47.5^a^			
*Evolution of the disease*														
Incidence of T2D 1000 pers-year	7.35	6.95	11.9	13.9	15.3	14.0	10.9	8.68	11.6	13.2	13.9	11.4	10.1	10.8^a^
Mortality rate 1000 pers-year	42.0	38.0	39.0	34.3	33.4	30.4	31.8	31.9	31.5	29.0	29.6	28.7	28.4	25.1^a^
Conversion rate from Prediabetes 1000 pers-year	65.5	84.0	90.3	97.5	105.0	80.9	57.8	44.6	59.6	62.9	59.8	43.1	41.1	48.1^a^
*Detection effect*														
HbA_1c_ testing frequency	24,003	30,271	45,129	58,630	67,533	72,035	73,395	76,854	82,610	90,949	105,214	118,260	138,073	145605^a^
HbA_1c_ at detection %	7.75	7.86	7.65	7.58	7.45	7.47	7.54	7.64	7.48	7.41	7.49	7.59	7.64	7.58^a^
Age at detection (year)	60.8	59.9	60.7	61.2	61.5	61.4	60.9	59.4	60.0	60.5	60.3	59.7	59.8	59.5^a^
Individuals with diabetes known as individuals with prediabetes %	0.97	1.70	2.33	3.25	4.19	3.65	2.75	2.12	2.93	3.29	3.30	2.41	2.23	2.66^a^

*T2D* type 2 diabetes

^a^ P < 0.0001

**Table 2:** Factors potentially involved in explaining changes in a population’s prevalence of diabetes

Categories of factors	Factors potentially involved
Individual-level risk factors	Age [5, 35–40], obesity [7–11, 13, 35–41], ethnicity [35, 42, 43], chronic disease (hypertension [44, 45], high triglycerides [44, 45], prediabetes [35, 46]), lifestyle (eating behavior [5, 6, 12, 14, 47], physical activity [5–7, 12, 14], smoking [5, 12, 13, 48], excessive alcohol consumption [14, 49, 50]), socio economic status [5, 51], low education [43, 51, 52], gestionnal diabetes [7, 35], intra uterin environnment [35], nutritional transition status [7] and diabetes familial history [5]
Environmental risk factors	Urbanization [5, 6, 12, 44, 53] environnmental pollution [35, 54–56] and rapid socioeconomic development [35]
Evolution of the disease	Increasing incidence rate [15, 17, 18, 23, 26, 27, 30, 32, 43, 57–63] decreasing mortality rate [15, 16, 18, 22–31, 64] and increasing conversion rate from prediabetes to diabetes [65]
Detection effect	Increase in number of people screened [22, 66] or diagnosed [57, 67] and decrease in people undiagnosed [9, 10, 57, 68, 69] or not screened [22], decreasing age at detection [36, 58, 70, 71] or increase in the prevalence of diabetes at earlier age [35, 45, 71], decreasing HbA_1c_ mean at detection and change in diagnostic criteria [35]
Global changes	Period effect [19] and birth cohort effect [19, 20]

### Individual-level risk factors

According to the literature review, the rise in a population’s prevalence of diabetes could be attributed to increases in that population’s prevalence of individual-level risk factors for the condition. Concurrently, we noted a marked increase in the prevalence of prediabetes from 2001 to 2014 (Table 1). Increases were also noted in the prevalence of obesity, hypertension, alcohol consumption, and immigration over the same period of time. During the study period, we also found the population was aging and that the prevalence of consumption of fruits and vegetables decreased.

However, the proportion of people reporting a physically active lifestyle, sedentary behaviours, tobacco smoking, having completed high school and university education, and being of low income evolved in directions that were opposite to the direction expected to be considered as a factor contributing to the increasing prevalence of diabetes.

### Environmental risk factors

The literature review has shown that some environmental risk factors such as the presence of environmental pollutants (such as nitrogen dioxide (NO_2_), particulate matter (PM), organic persistent pollutants and non-persistent pesticides), urbanization and rapid socioeconomic development could explain part of the increase in the prevalence of diabetes. In this study, the only factor that was possible to measure was the urbanization based on the proportion of people living in a rural area. This proportion decreased during the study period and evolved in a direction supporting that it may have influenced the increase in prevalence of diabetes in New Brunswick (Table 1).

### Evolution of the disease

Some factors characterizing the evolution of the disease, including survival time among individuals with diabetes, number of new cases of diabetes and conversion to diabetes from prediabetes population, could explain the increase in the prevalence of diabetes. In this study, we found that only two of these factors evolved in the direction expected if these factors were to explain the increase in prevalence of diabetes. The incidence rate increased from 2001 to 2014 and a higher increase in the incidence rate was seen around 2002 and 2005 (Table 1). The mortality rate of the population with diabetes decreased in the same period. In contrast, the conversion rate from prediabetes to diabetes could not explain the increase in prevalence of diabetes because it decreased during this period.

### Detection effect

**Fig. 1:**
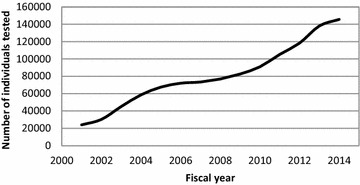
Number of individuals who received at least one HbA1c test by fiscal year

The literature review also suggested that a change in prevalence of disease could be attributed to changes in how the condition is identified. The detection effect could be related to an increase in the number of people being screened or diagnosed, an earlier detection of the disease and changes in diagnostic criteria. Accordingly we found that the number of people tested for HbA_1c_ increased from 2001 to 2014 and showed a higher increase around 2003 and 2010 (Fig. 1). Concurrently, the mean HbA_1c_ at detection decreased between 2001 and 2014 suggesting that people are being diagnosed earlier in the evolution of the disease (Table 1). It was also noted that the percentage of individuals with diabetes previously identified with prediabetes increased from 2001 to 2014, also suggesting that people are being detected at an earlier stage of their disease. The average age at detection also supports the presence of a detection effect since data suggest that people are being diagnosed at a younger age.

### Global changes

**Fig. 2:**
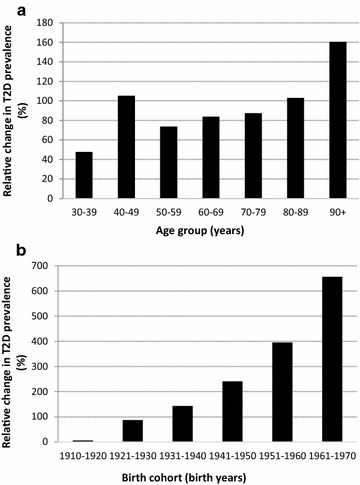
**a** Relative percentage of change in prevalence of type 2 diabetes by age groups (2001–2014). **b** Relative percentage of change in prevalence of type 2 diabetes by birth cohort group (2001–2014)

Changes in any or a combination of factors above may affect all of the population regardless of people’s age (period effect). Changes in factors could also be more pronounced in segments of the population, based on the year of birth (birth cohort effect). In this study, the presence of a period effect was supported by an increase in the prevalence of type 2 diabetes from 2001 and 2014 in each age group, with the 40–49 year old age group displaying a greater increase (Fig. 2a). The presence of a birth cohort effect was also suggested since the increase in prevalence of type 2 diabetes was considerably greater among the youngest birth cohort compared to the oldest birth cohorts (Fig. 2b).

## Discussion

The prevalence of type 2 diabetes has more than doubled in the past 15 years in New Brunswick. Although this prevalence change is relatively greater than changes noted elsewhere, this is consistent with the increase in prevalence in diabetes observed in recent years in other provinces and countries around the world [69, 72]. To help identify factors responsible for this increase, our literature review led to the identification of five categories of factors which together represent a comprehensive overview of factors that could explain a change in prevalence of diabetes. Our review included considerably more potential factors than what had been reported in other literature reviews [5–7, 35, 57, 73]. Guided by this inventory, we assessed the changes in nearly all factors suggested to potentially influence the prevalence of diabetes in New Brunswick. Through this work, we identified that changes in prevalence of diabetes in New Brunswick are likely attributed to a combination of numerous factors.

Among individual-level risk factors identified in the critical review, our analysis suggests that the presence of other conditions, such as the aging population, obesity, hypertension and prediabetes could contribute to explain the increase in prevalence of type 2 diabetes in New Brunswick. Furthermore, although immigration increased in NB, it is difficult to conclude that ethnicity might have an effect on the prevalence of type 2 diabetes in NB since no information is available on ethnic origin of immigrants in the databases used.

An increase in prevalence of obesity or body mass index has been linked to increases in the prevalence of diabetes in many other studies [8, 37–39, 68] and authors suggested it is the most important contributor to increases in prevalence of diabetes [8–10]. Consistent with changes in body composition of the population, we also noted that diet quality decreased over the study period. However, our data suggest that New Brunswickers became more physically active in the past 15 years, which does not concord with trends for obesity and diabetes. It has been suggested that this discrepancy may be attributable to measurement error associated with the use of self-reported measures [74]. Self-reported measures, such as physical activity levels, are suspected to have been influenced by an increase in social desirability possibly creating higher estimates over the years [74]. However, our results are consistent with the apparent increase in prevalence of a physically active lifestyle in the United States from 2001 and 2009, which seems to have had minimal impact on reducing the burden of obesity [75]. Among the potential for environmental risk factors to have influenced diabetes, we only had access to data relating to urbanization and these suggested that the transition from a predominantly rural to an urban population accompanied the increase in prevalence of diabetes in New Brunswick. This transition may mean that a greater proportion of the population may be exposed to obesogenic environments such as more sedentary work, higher use of car and public transport, television viewing and in short, lower levels of physical activity [6]. Although environmental pollution can be present in rural regions, it is also possible that urbanization has led to more exposure to pollutants. A meta-analysis has shown that the prolonged exposition to nitrogen dioxide (NO_2_), particulate matter less than 2.5 mm (PM_2.5_), and particulate matter less than 10 micrometers (PM_10_) was associated to type 2 diabetes incidence in cohort studies [55].

Changes in how diabetes evolves may also contribute to explaining a change in how the prevalence of disease increased in New Brunswick. Most particularly, we noted a decline in mortality rates of people with diabetes from 2001 to 2014, which is consistent with results from other studies [18, 22, 23, 26–28, 31]. The 40% decrease in mortality rates observed in our study over 14 years is similar to the 37.2% decrease in mortality witnessed in Ontario between 1996 and 2009 [29]. A longer survival period in the population with diabetes, likely explained by better treatment and control of the disease [48], therefore contributed to the increase in prevalence. An increase in the conversion rate from prediabetes to diabetes could also have explained an increase in diabetes prevalence [65]. However, this study shows a decrease in the conversion rate from prediabetes to diabetes, therefore suggesting either better prevention efforts in this population at risk or a relatively higher proportion of people identified with prediabetes through more screening.

As our data indicated, it is highly probable that part of the increase in prevalence of diabetes can be attributed to a detection effect whereby the number of people tested with HbA_1c_ rose markedly during the study period. Although the increase in testing was observed every year, sharper increases appeared to coincide with milestones such as the publication of the 2003 and 2008 guidelines encouraging type 2 diabetes screening [76, 77] and the identification of HbA_1c_ as a recognised diagnostic tool by the American Diabetes Association in 2010 [78]. The possibility of earlier detection of the disease is also supported by a decrease in HbA_1c_ levels and age at detection during the study period. The increase in testing and an early detection may also partly be explained by the study period corresponding with the introduction of financial incentives for physicians to offer recommended care to their patients with diabetes (2010), the implementation of an HbA1c tracking tool created to improve adherence to guidelines (2009) [79] and the Physician Practice profiles implemented in 2010 to aid physicians to identify at risk patients in New Brunswick. It should also be mentioned that although our analysis did not allow for the assessment of the relative contribution of each factor, another study suggested that improvements in detection of diabetes could explain as much as 25% of the increase in the prevalence of diabetes diagnosed since 1970 [10].

The fact that an increase in the prevalence of type 2 diabetes was observed in all age groups over the 15 years of study supports the presence of a period effect. The percentage of change in the prevalence of diabetes was nevertheless higher among the 40–49 years old, as it is possible that this group benefited from a higher detection effect than other age groups. The period effect could be attributed to a combination of any of the other factors identified in this study, including urbanization, increases in immigration and an increase in the detection. Other factors not measured, such as rapid socioeconomic development and increase in environmental pollution, could also be at play [19]. The data also presented evidence of a birth cohort effect as we observed higher increases in the prevalence of type 2 diabetes in the younger cohort groups. This could be explained by exposure early in life to some environmental factors related to an increase in obesity, as suggested by Soon et al. [19]. These results are consistent with others who demonstrated that steeper increases in the prevalence of diabetes in the youngest cohort parallel increases in the prevalence of obesity in the younger generations [19, 20]. The development of more obesogenic environment may affect younger people to a greater extent than other age groups since it represents a great proportion of their relative environmental exposure [19]. Further, another study reported that weight gain between 25 and 40 years old was associated with a higher risk of diabetes than a weight gain after 40 years old [80].

The present study has some limitations that need to be acknowledged. First, because of the descriptive design of the study, it was not possible to quantify and contrast the relative contribution of each factor. Second, the prevalence of diabetes and prediabetes could be underestimated due to the use of only one diagnostic method (HbA_1c_ test) in determining presence of disease and because HbA_1c_ was only endorsed as a diagnostic method after 2010. However, because the HbA_1c_ test had been in significant use prior to 2010 for the management of the disease [81], we hypothesise that most of the time a diagnosis of diabetes was followed by a HbA_1c_ test a short period after. In the same way, the detection effect measured in part by the number of people tested with a HbA_1c_ could be overestimated by the fact that the HbA_1c_ values for prediabetes or diabetes screening had not been identified before 2010 and we did not consider other screening tests such as fasting glucose and oral glucose tolerance test which were often used before 2010. However, HbA1c test has different sensitivity, specificity and utility than other diagnostic tests such as OGTT and fasting glucose [35] and because of that, it is advisable to use the same diagnostic test over time. Also, even though the HbA1c test is not universally accepted as a diagnostic tool [82] and may be affected by some individual conditions [83], it represents less individual variability than other tests and provides a better reflection of the glucose homeostasis in the long term [84]. Third, because it was not possible to distinguish the type of diabetes in the diabetes registry, it was not possible to exclude gestational diabetes cases and type 1 diabetes was excluded only on the basis of age. However, the 4% exclusion of potential type 1 diabetes individuals is close to the proportion of type 1 diabetes which is estimated to represent about 5-10% of cases of diabetes [85]. Fourth, it was not possible to measure some risk factors because of lack of data available, including gestational diabetes, intra uterin environnment, nutritional transition status, family diabetes history, environmental pollution, rapid socioeconomic development and high triglycerides. Fifth, our description of risk factors was performed concurrently to the change in prevalence of diabetes we were attempting to explain. Since diabetes has a long latency period, it may be appropriate to start describing those factors before 2001. This study also has strengths that need to be highlighted. This is the first study that describes an overview of factors that could explain the increase in prevalence of type 2 diabetes and used a population based analysis to measure almost all of those factors. The majority of analysis was done on the entire New Brunswick population and almost all the data was available for the 15 year period.

## Conclusions

In conclusion, this study presents a comprehensive overview of potential factors that could explain the change in the prevalence of type 2 diabetes. This study shows that for the past 15 years, the prevalence of diabetes in New Brunswick has increased considerably and this increase could be explained by many factors including some individual-level and environmental risk factors, the detection effect, the evolution of the disease and global changes. However, the increasing prevalence of diabetes observed may not be as impressive as it appears due to the significant influence of the detection effect and the dramatic decrease in mortality. A better understanding of factors potentially responsible for the increase in type 2 diabetes can assist in making informed decisions about diabetes programs and policies. This study may be used as a template for other countries or provinces to identify factors that could explain the increase in the prevalence of diabetes in their respective jurisdictions. More research is needed to measure the relative contribution of each factor on the increase in prevalence of diabetes by measuring each factor directly at the individual level and to evaluate the change in risk factors earlier in the evolution of the disease.
